# Latinx youth’s and parents’ covid-19 beliefs, vaccine hesitancy and vaccination rates: Longitudinal associations in a community sample

**DOI:** 10.1371/journal.pone.0307479

**Published:** 2024-07-24

**Authors:** Namoonga M. Mantina, Maiya G. Block Ngaybe, Katharine H. Zeiders, Kayla M. Osman, Ada M. Wilkinson-Lee, Antoinette M. Landor, Lindsay T. Hoyt

**Affiliations:** 1 Department of Health Promotion Sciences, Mel & Enid Zuckerman College of Public Health, University of Arizona, Tucson, Arizona, United States of America; 2 Norton School of Human Ecology, University of Arizona, Tucson, Arizona, United States of America; 3 Department of Mexican American Studies, University of Arizona, Tucson, Arizona, United States of America; 4 Department of Human Development and Family Science, University of Missouri, Columbia, Missouri, United States of America; 5 Department of Psychology, Fordham University, Bronx, New York, United States of America; UT Southwestern: The University of Texas Southwestern Medical Center, UNITED STATES OF AMERICA

## Abstract

**Introduction:**

The Latinx population has the second highest COVID-19 death rate among racial/ethnic groups in the United States and less than half of Latinx youth aged 5–17 years old completed their COVID-19 primary vaccination series as of September 2022. COVID-19 vaccine misinformation detrimentally impacts vaccination rates. In this study, we examined factors that predicted Latinx youth COVID-19 vaccine hesitancy and vaccination status.

**Methods:**

A community-based sample of 290 Latinx parent and adolescent dyads from a Southwestern metropolitan area of the United States who were recruited to complete an online survey at baseline at T1 (August 2020 –March 2021) and one year later. We tested a longitudinal mediation model in which we examined individual and family factors that would predict youth COVID-19 vaccine hesitancy and vaccination status over time.

**Results:**

Youth’s pandemic disbelief (i.e., the belief that the COVID-19 pandemic is a conspiracy or not real) predicted greater youth’s COVID-19 vaccine hesitancy, and in turn, a lower likelihood of youth’s COVID-19 vaccination. Youth’s pandemic disbelief also predicted greater parent’s vaccination hesitancy which, in turn, predicted greater youth’s vaccination hesitancy and a lower likelihood of COVID-19 vaccination. Parents’ pandemic disbelief predicted their own COVID-19 hesitancy, but not youth hesitancy.

**Discussion:**

Our study findings provide initial evidence that general pandemic disbelief was a significant driver of vaccine hesitancy and vaccination among Latinx families. The study contributes to the limited research investigating COVID-19 vaccination in the Latinx community and among Latinx youth, further aiding how COVID-19 vaccine disparities can be mitigated among racial/ethnic populations.

## Introduction

The Hispanic/Latino/Latinx [1] community makes up 19% of the United States population yet accounted for 24.5% of SARS-CoV-2 (COVID-19) cases, second highest only to non-Hispanic Whites [1, 2]. Although 84% of Latinx adults over 18 years had completed the primary series of COVID-19 vaccines by March 2023 [3], this community has been impacted by systemic inequities that have resulted in disproportionate health outcomes. The Latinx population has the second highest COVID-19 death rate among racial/ethnic groups in the US [2]. Although vaccine hesitancy, defined as the delay, refusal, or reluctance to get vaccinated, existed before the COVID-19 pandemic [4], COVID-19 detrimentally impacted the administration of longstanding routine childhood immunizations globally [5–7]. In the US, the COVID-19 pandemic was associated with increases in general vaccine hesitancy and childhood vaccine hesitancy among parents [8]. Additionally, the COVID-19 pandemic exacerbated vaccination misinformation and conspiracy theories [9], in part because of political rhetoric that introduced a new politicization context for vaccination hesitancy and behavior [10]. Indeed, political affiliation in parents has been shown to be related to parents’ intention to vaccinate their child against COVID-19 [11], but very little work has examined the relation of COVID-19 vaccine-related conspiracy theories and politicization in youth alongside parental factors. Given this, the current study explored the pathways linking parents’ and youth’s disbelief of COVID-19 (i.e., the belief that the COVID-19 pandemic is a conspiracy or not real) early in the pandemic (from August 2020) to vaccine hesitancy and vaccination rates one year later (2021) among a community-based sample of Latinx families.

COVID-19 vaccines were approved under Emergency Use Authorization for children ages 5 to 17 years old by October 2021 [12–14]. However, COVID-19 vaccination rates among adolescents were lower than desired. In September 2022, only 47.2% of youth 5–17 years had received at least one COVID-19 dose and 43.3% received two or more doses [15]. There are many factors that relate to *children’s* vaccination rates, with the most salient being parental vaccination decisions, but other factors include safety concerns, fear of long-term side effects, and potential negative reactions to the vaccine [16]. Parents with lower income have also reported greater hesitancy about their child receiving a COVID-19 vaccine compared to those with higher income [17, 18]. Parental COVID-19 vaccine hesitancy has been found to vary across racial/ethnic groups. Latinx and Black parents have reported more hesitancy about their child receiving the COVID-19 vaccine compared to White parents [17, 19]. Factors associated with greater vaccine hesitancy among racial/ethnic populations include perceived risk of COVID-19, vaccine beliefs, vaccination history, and concerns for safety, efficacy of the vaccine, medical mistrust, and history of racial discrimination [19, 20].

Notably, family decisions about vaccinations are not one-directional—children can also influence the values, beliefs, and attitudes of their parents [21]. Although parents are the key decision makers for their child’s medical care, adolescents are not always passive observers in these processes. For instance, in a study examining parents and sons’ vaccinations, researchers found that when parents included adolescents in decisions about the human papillomavirus vaccination (HPV), there was greater open communication, shared decision making and increases in adolescents’ vaccination uptake [22, 23].

Outside of familial influences, additional societal factors can influence vaccine uptake, including misinformation, conspiracy theories, and political climate. General childhood vaccine misinformation has been found to relate to parental vaccine hesitancy and, as a result, has been shown to influence vaccine attitudes with conservative individuals more likely to believe in conspiracy theories about vaccines than liberal individuals [24]. More conservative parents are less likely to hold pro-vaccine beliefs and are less likely to vaccinate their children for recommended childhood vaccines [25, 26]. The politics, misinformation, vaccine rumors, and conspiracy theories surrounding the COVID-19 vaccine have been found to relate to COVID-19 vaccine hesitancy [27–30] across multiple racial and ethnic groups [19]. Further, parents who are more prone to believe in conspiracy theories are more hesitant in getting their children vaccinated for COVID-19 [31]. Within immigrant communities and among foreign-born Hispanic individuals specifically, increasing fears and concerns relating to documentation and deportation have also been found to impact COVID-19 vaccinations [19, 30–32]. Much of this prior research has been conducted outside the US, highlighting global patterns. This study focuses on these factors within a southwestern US state (that has a high percentage of Hispanic/Latinx immigrants), considering the local context and political climate around vaccination. Although it is critical to understand parental beliefs about adolescent COVID-19 vaccination, it is also imperative to study young people’s experiences. There is, however, a dearth of research investigating adolescents’ conspiracy beliefs about the COVID-19 pandemic. One study found that 14% of their sample of German adolescents endorsed at least one COVID-19 conspiracy belief [33]. A qualitative study revealed several beliefs held among a sample of South African youth such as COVID-19 being human-made to harm people [34]. Given the prominence of COVID-19 conspiracy theories and misinformation, understanding the COVID-19 beliefs among parents and adolescents may be essential for understanding the disparities seen in adolescent COVID-19 vaccination rates.

### The current study

The current study aimed to provide descriptive information about the COVID-19 vaccination rate and COVID-19 vaccine hesitancy among a community-based sample of Latinx youth and parents. Additionally, we tested a longitudinal mediation model in which we examined factors that would predict youth COVID-19 vaccine hesitancy and vaccination status over time. Specifically, we examined how youth and parents’ (dis)beliefs about the pandemic and individual (i.e., youth age; parent/youth gender and nativity) and family (i.e., family income) characteristics related to youth COVID-19 vaccination hesitancy and COVID-19 vaccination status one year later. Given that vaccine attitudes have been found to predict vaccination [35, 36], we tested for mediation, hypothesizing that beliefs, individual, and family characteristics would relate to parent/child vaccination via vaccination hesitancy.

## Methods

### Procedure

Data came from The Hijos Project, a large longitudinal research project on the experiences, stressors, and well-being of Latinx families with adolescent children during the COVID-19 pandemic. From August 1, 2020, to March 30, 2021, families were recruited through social media and local organizations. Families were eligible if one parent (or caregiver) and one adolescent, between the ages of 11 to 15 years, were of Latinx background. Families were screened for eligibility through follow-up phone calls in which bilingual study personnel validated the name of the parent and adolescent, and the adolescent’s age. Eligible, consenting parents and assenting adolescents received a link to complete a 30-to-45-minute online survey available in English or Spanish. Given the interest in both parent and youth vaccine status, the current study utilized data from families where both the adolescent and the parent completed the study at Time 1 (T1) (*N* = 290 parent-child dyads).

Families were followed up at approximately one year (Parent: M = 1.08 years, SD = .10 years; Youth: M = 1.09 years, SD = .10 years) after T1. Approximately 79% of parents and 78% of adolescents from T1 completed an online survey at Time 2 (T2), which occurred from November 1, 2021, to April 30, 3022. Parents and youth received a $25 and $10 e-gift card, respectively at T1 and a $30 and $25 e-gift card at T2. The University Institutional Review Board (IRB #2005739923; IRB #2108136675) approved the study.

### Sample characteristics

At T1, parents were, on average, 39.8 years old (SD = 6.65); 90% of parents identified as women/female, and 9% as men/male (Table 1). Most parents were US-born (57.9%) and completed the survey in English (69%); among those who were foreign-born, the majority (41%) were born in Mexico and came to the US when they were, on average, 18.9 years old (SD = 10.2). Most parents identified as Mexican American (39.7%), reported ‘some college, vocational or technical school’ (20.7%) and reported a yearly household income of $50,000 or lower (56.9%). Adolescents were, on average, 13.7 years old (*SD* = 1.42) at T1; 50.3% of adolescents identified as boys/males, and 47.6% as girls/females. Almost all adolescents were US-born (95.2%) and chose to complete the survey in English (97.9%); those who were foreign-born came to the US when they were, on average, 7.4 years old (SD = 3.52) and the majority were born in Mexico. Most adolescents identified their ethnic background as Mexican American (58.6%).

**Table 1:** Characteristics of parent-adolescent dyad sample (n = 290).

Characteristics	Parent		Adolescent	
	n	(%)	n	(%)
**Age**, years (mean, [SD])	39.8	[6.65]	13.7	[1.42]
**Gender**				
Woman/girl/female	261	(90.3)	138	(47.6)
Man/boy/male	26	(9.0)	146	(50.3)
Genderqueer/non-conforming	1	(0.4)		
Non-binary	1	(0.4)	5	(1.7)
Trans girl/female			1	(0.3)
**Ethnicity** (multi-select options)				
Mexican American		(39.7)		(58.6)
Mexican		(27.6)		(9.7)
Hispanic		(22.4)		(17.9)
Chicano		(4.8)		(2.4)
Latino/Latinx		(4.5)		(7.9)
Other		(1.0)		(2.8)
**Country of Birth**				
US-born	168	(57.9)	276	(95.2)
Foreign-born	122	(42.1)	14	(4.8)
Mexico	119	(41.0)	12	(4.1)
Another Country	3	(1.0)	2	(0.7)
Age upon US arrival (mean, [SD])	18.9	[10.2]	13.7	[1.42]
**Highest level of Education**				
6th Grade			13	(5.7)
7th Grade			42	(18.5)
8th Grade			37	(16.3)
8th Grade			57	(25.1)
10th Grade			38	(16.7)
11th Grade			36	(15.9)
12th Grade			4	(1.8)
Elementary–some high school	31	(13.4)		
High school diploma/GED	37	(16.0)		
Some college, vocational, technical	48	(20.7)		
Vocational or technical graduate	18	(7.8)		
Associate degree	21	(9.1)		
Bachelor’s degree	36	(15.5)		
Master’s degree	34	(14.7)		
Doctorate/advanced degree/some work	4	(1.7)		
**Family Income**				
$10,000 or less	40	(13.8)		
$10,001–$20,000	20	(6.9)		
$20,001–$30,000	19	(6.6)		
$30,001–$40,000	39	(13.4)		
$40,001–$50,000	47	(16.2)		
$60,001–$60,000	26	(9.0)		
$60,001–$70,000	22	(7.6)		
$70,001–$80,000	14	(4.8)		
$80,001–$90,000	22	(7.6)		
$90,001–$100,000	10	(3.4)		
Over $100,000	31	(14.1)		
**Survey Language Completion**				
English		(69.0)		(97.9)
Spanish		(31.0)		(2.1)

### Measures

#### COVID-19 conspiracy beliefs (T1)

Similar to other studies who created new measures during the COVID-19 pandemic [37], we utilized a single item to ask parents and youth about their beliefs regarding the COVID-19 pandemic at T1. Parents were asked to rate their agreement with the statement, “The COVID-19 pandemic is a conspiracy” and youth rated their agreement with the statement, “The COVID-19 pandemic is fake or not real.” Responses ranged from 1 = *Strongly Disagree* to 5 = *Strongly Agree*.

#### Individual and family predictors (T1)

Parents were asked to report on their total family income. To assess income, response categories ranged from “less than $10,000” and “$150,000 or higher.” Adolescents reported on their gender, age, and nativity. Youth were asked to report their gender identity with the following question, “What is your current gender identity?” with the option to choose between 6 different gender identity responses as well as the option to write in their own response. Gender was coded as 0 (boy/male/transgender boy/male), 1 (girl/female/transgender girl/female), and non-binary youth (n = 5) were coded as missing. To assess nativity, both parents and adolescents were asked to specify their country of birth. Nativity was coded as 0 (foreign-born) and 1 (US-born).

#### Family COVID-19 hospitalizations or deaths (T2)

We accounted for families’ COVID-19 hospitalizations and deaths during the pandemic as these may act as proxies to perceived risk and relate to their own COVID-19 risk-related health beliefs. Parents were asked to report their familial experiences with COVID-19 related to hospitalization and family deaths with two items at T2. They reported on whether “Someone in the family was hospitalized for COVID-19” and if “Someone in the family died from COVID-19” (0 = No and 1 = Yes). A sum score was computed to indicate the total number of experiences, which could range from 0 to 2.

#### General vaccine hesitancy (T2)

Parents were asked to complete an adapted Vaccine Hesitancy Scale adapted from the Vaccine Hesitancy scale [38]. Using 7 items, parents rated their agreement with statements related to their perceptions of general children’s vaccines (e.g., “New vaccines carry more risks than older vaccines”). Responses ranged from 1 = *Strongly Disagree* to 5 = *Strongly Agree*. A mean was computed to create a total vaccine hesitancy score; higher scores reflected greater levels of hesitancy. The scale demonstrated strong reliability (α = .92).

#### COVID-19 vaccine hesitancy (T2)

Both parents and youth were asked to complete an adapted version of the Vaccine Hesitancy Scale [39] where items were specific to COVID-19 vaccines. Parents and youth rated their agreement with statements related to their perceptions of the COVID-19 vaccines (e.g., “I am concerned about serious adverse effects of the COVID-19 vaccine”). Responses ranged from 1 = *Strongly Disagree* to 5 = *Strongly Agree*. A mean was computed to create a total COVID-19 vaccine hesitancy score. Higher scores reflect greater levels of hesitancy. The scale demonstrated strong reliability for both parents (α = .92) and youth (α = .91).

#### COVID-19 vaccination (T2)

Both parents and youth were asked to report whether they had received the COVID-19 vaccine with a single item (i.e., “Have you gotten the COVID-19 vaccine?”; 0 = *No* and 1 = *Yes*). Those who responded “No” were asked “Are you planning on getting the COVID-19 vaccine?” (0 = *No* and 1 = *Yes*).

### Statistical analysis

**Fig 1:**
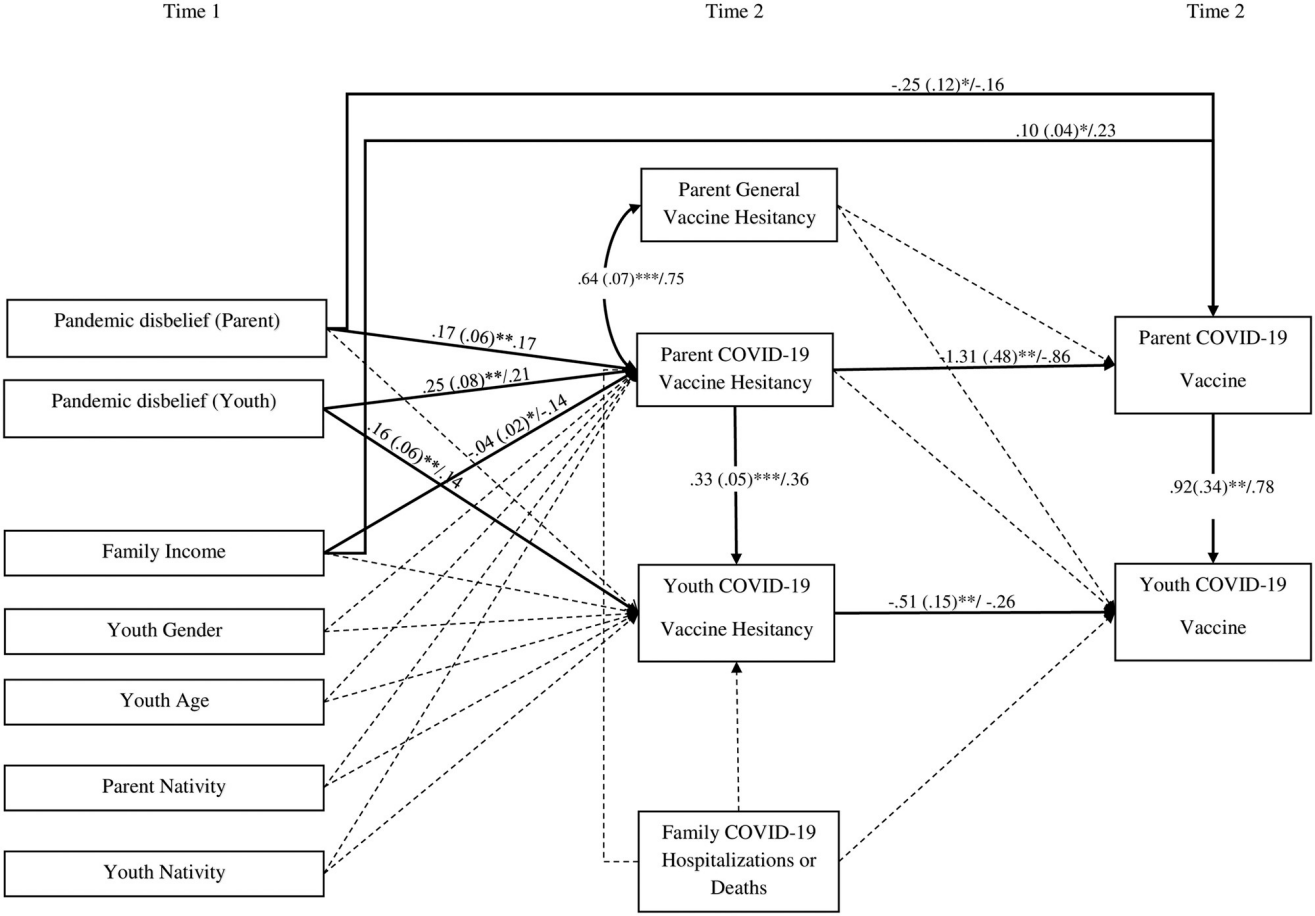
Path model linking COVID-19 disbelief to youth COVID-19 vaccination via COVID-19 vaccine hesitancy. Youth and parent pandemic disbelief, individual, and familial factors at Time 1 (August 2020 to March 2021) predicting youth and parent COVID-19 vaccine hesitancy and COVID-19 vaccination status 1 year later (Time 2, November 2021 to April 2022), controlling for parent general vaccination hesitancy and family COVID-19 hospitalizations and deaths. Model fit: χ2 (3) = 1.65, ns; CFI = 1.00, RMSEA = .00, SRMR = .01. * p < .05, ** p < .01, *** p < .001. Paths bolded are significant; dashed paths are non-significant. Unstandardized coefficients (and standard errors) are first reported; standardized coefficients are presented after the dash. Note that all paths to the following constructs are log odds: Parent COVID-19 Vaccine and Youth COVID-19 Vaccines. Youth gender coded 0 = boy; 1 = girl, Parent and youth nativity coded 0 = born outside of US, 1 = born in the US. Youth COVID-19 Vaccine r^2^ = .68; Parent COVID-19 Vaccine r^2^ = .56; Parent COVID-19 Vaccine Hesitancy r^2^ = .13; Youth COVID-19 Vaccine Hesitancy r^2^ = .20.

Analyses were performed in SPSS V 28.0 and MPlus Version 8.1 [40]. For path models, multiple fit indices—chi-squared test, comparative fit index (CFI), root mean square error of approximation (RMSEA), and standardized root mean square residual (SRMR)—were used to assess model fit; good model fit is reflected by a non-significant chi-squared test, CFI greater than .95, RMSEA less than .05, and SRMR less than .05 [41]. The hypothesized model (Fig 1) was specified in MPlus. Specifically, T1 youth COVID-19 vaccine status was regressed on T2 parent COVID-19 vaccine status and T2 parent and youth COVID-19 vaccine hesitancy. T2 Parent COVID-19 vaccine status was regressed on T2 parent and youth COVID-19 vaccine hesitancy. Given that both T2 youth and parent COVID-19 vaccine status were dichotomous variables, theses estimations were logistic regressions (and thus, coefficients represent log odds). T2 Parent COVID-19 vaccine hesitancy was regressed on T1 parent and youth pandemic disbelief, as well as T1 individual and family predictors (i.e., family income, youth gender, youth age, parent, and youth nativity). T2 youth COVID-19 vaccine hesitancy was regressed on T2 parent vaccine hesitancy, T1 parent and youth pandemic disbelief and T1 individual and family predictors. Given that we were testing mediation, all direct paths were specified as well (e.g., youth and parent COVID-19 vaccines status regressed on parent and youth pandemic disbelief). Additionally, covariates were included in the model; specifically, paths from T2 parental general vaccine hesitancy to T2 parent and youth COVID-19 vaccine status was specified and paths from T2 family COVID-19 hospitalizations/deaths to T2 youth and parent vaccine hesitancy and youth COVID-19 vaccine status were specified. Missing data were accounted for using full information maximum likelihood (FIML) [42] and mediation was tested using the multivariate delta method [43].

## Results

The first set of descriptive results includes all participants with complete data on T2 vaccination status (i.e., T2 parent-adolescent dyad sample), which includes 228 youth and 231 parents. Nearly 80.9% of these youth reported receiving at least one vaccination for COVID-19 at T2 (Table 2); of those who reported not being vaccinated, 45.5% reported that they intend to receive the vaccination. Most parents (88.4%) reported receiving at least one COVID-19 vaccination (Table 2); of those parents who reported not being vaccinated, 33.3% reported that they intended to be vaccinated. We also examined family dyad vaccination rates (i.e., parent and youth within the same family); 77.9% of families included vaccinated youth and parents, 7.9% of families reported neither the parent nor child were vaccinated, 3.5% of families reported only the parent was vaccinated, and finally, 11.4% of families reported that only the child was vaccinated. As for COVID-19 vaccine hesitancy, both parents and youth reported relatively low hesitancy (*M* = 2.18, *SD* = 0.9; *M* = 2.16, *SD* = 1.0 respectively). COVID-19 vaccine hesitancy did not differ across US and foreign-born parents, *t* = .77(229), *p* = .44, nor did they differ across US and foreign-born youth, *t* = .32 (227), *p* = .09.

**Table 2:** Percentages of COVID-19 vaccination rates among T2 parent-adolescent dyad sample.

	**Youth Vaccinated (n, %)**	
**Parent Vaccinated (n, %)**	**Yes** (184, 80.7%)	**No** (44, 19.3%)
**Yes** (204, 88.3%)	175 (77.1%)	8 (3.5%)
**No** (27, 11.7%)	26 (11.5%)	18 (7.9%)

Note. Youth total T2 *n* = 228; Parent total T2 *n* = 231.

In terms of differences by nativity, COVID-19 vaccine rates were higher among US-born youth (81.6%) compared to foreign-born youth (63.6%), but not statistically different, χ2 (1) = 2.16, p = .14 (Table 3). There was also no significant difference in youth vaccination status by parents’ nativity, χ2 (1) = .14, p = .71; that is, youths’ COVID-19 vaccine rates were 79.8% for youth whose parent was born in the US and 81.8% for youth whose parent was born outside of the US. Among parents, 87.2% US-born parents and 89.8% of foreign-born parents reported COVID-19 vaccination and these percentages were not significantly different χ2 (1) = .36, p = .55.

**Table 3:** Percentages of T2 COVID-19 vaccination rates by nativity among the T2 parent-adolescent dyad sample.

**Youth vaccination status**	**Youth born in US**	**Youth born out of US**	**Pearson chi-squared p-value**
	n *(%)*	n *(%)*	
No, COVID-19 vaccination	40 *(18*.*4)*	4 *(36*.*4)*	
Yes, COVID-19 vaccination	177 *(81*.*6)*	7 *(63*.*6)*	0.14
**Youth vaccination status**	**Parents born in US**	**Parents born out of US**	**Pearson chi-squared p-value**
	n *(%)*	n *(%)*	
No, COVID-19 vaccination	26 *(20*.*2)*	18 *(18*.*2)*	
Yes, COVID-19 vaccination	103 *(79*.*8)*	81 *(81*.*8)*	0.71
**Parent vaccination status**	**Parents born in US**	**Parents born out of US**	**Pearson chi-squared p-value**
	n *(%)*	n *(%)*	
No, COVID-19 vaccination	17 *(12*.*8)*	10 *(10*.*2)*	
Yes, COVID-19 vaccination	116 *(87*.*2)*	88 *(89*.*8)*	0.55

Note. Youth total T2 *n* = 228; Parent total T2 *n* = 231.

Table 4 presents correlations and descriptive information among study constructs.

**Table 4:** Correlations and descriptive statistics for study variables.

	1.	2.	3.	4.	5.	6.	7.	8.	9.	10.	11.	12.	13.
1. Family Income	--												
2. Parent Nativity	.16**	--											
3. Youth Nativity	.01	.23**	--										
4. Youth Gender	.08	.*02*	.02	--									
5. Youth Age	.08	.*04*	-.04	.07	--								
6. Parent Pandemic Disbelief (T1)	-.10	*-*.37**	-.07	.01	.07	--							
7. Youth Pandemic Disbelief (T1)	-.08	*-*.*00*	-.01	-.14*	-.00	.25**	--						
8. COVID-19 Hospitalizations/Deaths (T2)	.08	.*04*	.00	.04	-.04	-.14*	-.02	--					
9. Parent General Vaccine Hesitancy (T2)	-.11	-.06	-.01	-.12	.14*	.14*	.22**	-.03	--				
10. Parent COVID-19 Vaccine Hesitancy (T2)	-.16*	-.05	-.02	-.10	.11	.22**	.27**	.01	.79**	--			
11. Youth COVID-19 Vaccine Hesitancy (T2)	-.12	.05	-.02	-.12	.04	.04	.24**	.07	.33**	.39**	--		
12. Parent COVID-19 Vaccine Status (T2)	.17	-.04	.04	.05	-.13	-.20**	-.14*	-.02	-.26**	-.48**	-.22**	--	
13. Youth COVID-19 Vaccine Status (T2)	.17	-.03	.10	.14*	.01	-.18**	-.24**	.06	-.25**	-.38**	-.36**	.45**	--
Mean	--	--	--	--	13.17	1.64	1.46	.50	1.97	2.16	2.18	--	--
Standard deviation	--	--	--	--	1.42	.96	.84	.80	.89	1.00	.90	--	--
Minimum value	--	--	--	--	11.00	1.00	1.00	.00	1.00	1.00	1.00	--	--
Maximum value	--	--	--	--	15.00	5.00	5.00	2.00	4.71	5.00	5.00	--	--

Note. Due to missing data from Time 1 to Time 2 of the study, sample sizes varied from 229 to 290. Gender was coded 0 = boy/male, 1 = girl/female. Nativity was coded 0 = foreign-born, 1 = US-born. Vaccine Status was coded 0 = no, 1 = yes.

*p < .05,

**p < .01.

The column numbers correspond with the row numbers and labels. All coefficients reflect bivariate correlations.

Fig 1 presents path modeling analysis results using the full sample of 290 parent-adolescent dyads, accounting for missing data with FIML; both unstandardized and standardized estimates are presented in the figure for linear regressions; for paths that were logistic regressions, both log odds and standardized log odds are presented. The model demonstrated good fit, χ2 (3) = 1.65, ns; CFI = 1.00, RMSEA = .00, SRMR = .01., and suggested that youth’s pandemic disbelief at T1 predicted youth’s and parents’ greater COVID-19 vaccine hesitancy at T2. Parents’ pandemic disbelief predicted their own COVID-19 hesitancy, but not youth’s hesitancy. At T2, youth’s COVID-19 vaccine hesitancy predicted youth’s own COVID-19 vaccination status, and parent’s COVID-19 vaccine hesitancy predicted parent’s COVID-19 vaccination status. Additionally, parents’ COVID-19 vaccine hesitancy predicted youth’s greater COVID-19 vaccine hesitancy; and parent’s COVID-19 vaccination predicted youth’s COVID-19 vaccination in the expected direction. Greater T1 family income related to lower T2 COVID-19 vaccination hesitancy among parents and a higher likelihood of parents’ T2 COVID-19 vaccination; no other family factor was statistically significant.

Mediational tests revealed five significant mediational pathways. First, youth’s T2 COVID-19 vaccine hesitancy significantly mediated the link between youths’ T1 disbelief and youth’s T2 COVID-19 vaccination, *b*_*indirect effect(ie)*_ = -.08, *SE* = .04, *p* < .05. Additionally, parents’ and youths’ T2 COVID-19 vaccine hesitancy significantly mediated the link between youth’s T1 pandemic disbelief and youth’s T2 COVID-19 vaccination, *b*_*ie*_ = -.04, *SE* = .02, *p* < .05. Similarly, parents and youths’ vaccine hesitancy significantly mediated the link between parents’ T1 pandemic disbelief and youths’ T2 COVID-19 vaccinations, *b*_*ie*_ = -.03, *SE* = .01, *p* < .05. In terms of parents’ COVID-19 vaccination rates, parents’ T2 vaccine hesitancy significantly mediated the link between parents T1 disbelief and their T2 COVID-19 vaccinations, *b*_*ie*_ = .23, *SE* = .11, *p* < .05 and the link between youth’s T1 pandemic disbelief related and parents’ T2 COVID-19 vaccination, *b*_*ie*_ = -.32, *SE* = .15, *p* < .05.

## Discussion

Youth COVID-19 vaccination rates have been disproportionately low, triggering a critical need to understand the factors that influence their vaccination uptake to ensure adolescents continue to be protected from COVID-19. This study contributes to the limited research investigating COVID-19 vaccination in the Latinx community. Our results revealed that Latinx youth in our sample were almost twice as likely to be vaccinated than estimates from national sample of Latinx youth (80.7% vs 43.5%) [15]. Further, our study provides initial evidence that general pandemic disbelief was a significant driver of vaccine hesitancy and vaccination among Latinx parents and youth, and that youth and parent pandemic disbelief together help us understand vaccine hesitancy within families. Our findings underscore the importance of larger socio-historical contexts in vaccination efforts and the specific need to address COVID-19 myths and misinformation to boost vaccination uptake and reduce vaccination disparities.

Within our sample of Latinx families, over 88% of parents and 80% youth received at least one dose of the COVID-19 vaccine. These results are higher than previously reported vaccination rates in Latinx population, particularly with studies that compare vaccination rates across multiple races [44]. Interestingly, compared to studies that research the Hispanic population exclusively, our high COVID-19 vaccination results are consistent [45]. It is plausible that sampling bias due to convenience sampling may be underpinning the high COVID-19 vaccination results we observed.

One of the most prominent findings of the study was that pandemic disbelief emerged as a driving factor in COVID-19 vaccine hesitancy, and ultimately, vaccination rates within Latinx families. For both youth and parents, their own pandemic disbelief predicted their likelihood of receiving the COVID-19 vaccine via their own COVID-19 hesitancy. This finding is consistent with previous studies which demonstrated the mediating effect of vaccine hesitancy on the relationship between the quality of information or conspiracy theories and the outcome of vaccination against COVID-19 [30, 46].

Interestingly, in addition to youth pandemic disbelief predicting their own vaccine hesitancy, youth’s disbelief also predicted their *parents’ vaccine hesitancy* (but parents’ pandemic disbelief did not predict youth hesitancy). These results lend support to the bidirectional influences within a parent-child relationship regarding vaccination, and the unique role of adolescents in shaping familial views on vaccinations. Parents are often thought of as the key decision maker for children under the age of 18, and although the final decision may rest with them, our findings suggest that their child’s perspective, particularly around disbelief, may be part of this decision-making process. Indeed, research has shown that parents in general who consider their child’s perceived desire to get vaccinated were more likely to want their child vaccinated [47]. And for our study specifically, in reference to Latinx cultural values related to familism and the importance of family members in decisions making, it may be that Latinx parents are taking cues from their children related to the COVID-19 pandemic—whether their child believed it was fake or not real—and then forming their own opinions and hesitancies, which in turn predicted their children’s hesitancy and both family members vaccination decision. These findings highlight the need to not only address misinformation and the way that it shapes vaccine hesitancy and rates, but to do so in age-specific and culturally appropriate ways to address COVID-19 vaccination disparities.

There are a few additional findings worth noting. First, it was notable that family income was the only demographic factor that related to parent vaccine hesitancy and parent vaccination status. This finding was consistent with other research suggesting that greater income was related to lower vaccine hesitancy (in parents) and a greater likelihood of COVID-19 vaccination (in parents) [17]. We found that nativity was unrelated to COVID-19 vaccine hesitancy, and this is in line with some previous work that suggests no difference in vaccine hesitancy between foreign-born Hispanics and US-born Whites [48, 49]. Although our study sample was entirely Hispanic/Latinx, it is plausible that other factors may offset differences in COVID-19 hesitancy between US-born and foreign-born participants.

In summary, the study utilized a community-based Latinx sample–a population historically underrepresented in research–and explored the ways disbelief and vaccine hesitancy related to COVID-19 vaccination rates. Using a longitudinal design, we examined prospective changes over time in both parents and adolescents, facilitating an understanding of COVID-19 vaccination behavior and attitudes within a family context. This is one of the first known studies that explores adolescents’ COVID-19 beliefs and their association with COVID-19 vaccination hesitancy in a sample of US-based Latinx adolescents.

Study findings should be interpreted with some limitations in mind. First, we did not inquire about the number of shots they received in our vaccination measure. This limits our ability to assess any differences in vaccine initiation versus vaccine series completion for full protection from COVID-19. Similarly, the study did not access families’ to COVID-19 vaccination, a reported barrier to COVID-19 vaccination uptake [50]. Additionally, the one-item pandemic disbelief measure may not have captured the full scope or subtleties of COVID-19 pandemic perceptions or beliefs. Indeed, previous research has tied political affiliation and religious beliefs to COVID-19 beliefs [51]. Understanding the ways that beliefs, misinformation, and politics intersect is needed. Related, we did not assess explicit decision-making processes among adolescents and parents. Given that adolescence is a period in which autonomy increases in the context of greater extrafamilial relationships and influence, future research should examine the ways these co-occur to impact parent-child decision making around vaccines. Finally, we relied upon a convenience sample of Latinx families from Southern Arizona. Given the geographic location, cultural influences that may be unique to this region, and that Hispanic/Latinx experiences are not monolithic, our results may not be generalizable across the broader population of Latinx communities across the US and globally.

Despite the limitations, this study contributes to our understanding COVID-19 vaccination hesitancy and vaccination uptake within a Latinx sample, further aiding how COVID-19 vaccine disparities can be mitigated among ethnic populations. Our results highlight the affect youth beliefs have on parents’ vaccination attitudes for themselves and their children. School or community-based interventions may be useful in educating youth on disease risk reduction and prevention strategies, with special attention to parent-child relationships and addressing misinformation. Additionally, Latinx families are known to have strong family ties, and within the context of a family social network, there may be opportunities to intervene with key individuals that may provide benefit to the whole family network. Such factors are important when designing future interventions, policies, and health messaging to promote and ensure effective vaccination uptake in the future.
